# Molecular diversity within the genus *Laeonereis* (Annelida, Nereididae) along the west Atlantic coast: paving the way for integrative taxonomy

**DOI:** 10.7717/peerj.11364

**Published:** 2021-05-27

**Authors:** Bruno R. Sampieri, Pedro E. Vieira, Marcos A. L. Teixeira, Victor C. Seixas, Paulo R. Pagliosa, Antonia Cecília Z. Amaral, Filipe O. Costa

**Affiliations:** 1 Centro de Biologia Molecular e Ambiental, Universidade do Minho, Braga, Portugal; 2 Museu de Zoologia, Universidade Estadual de Campinas, Campinas, São Paulo, Brazil; 3 Institute of Science and Innovation for Bio-Sustainability (IB-S), Universidade do Minho, Braga, Portugal; 4 Programa de Pós-Graduação em Ecologia, Instituto de Biologia, Universidade Federal do Rio de Janeiro, Rio de Janeiro, Brazil; 5 Laboratório de Biodiversidade e Conservação Marinha, Universidade Federal de Santa Catarina, Florianopólis, Santa Catarina, Brazil; 6 Departamento de Biologia Animal, Universidade Estadual de Campinas, Campinas, São Paulo, Brazil

**Keywords:** Polychaeta, Multi-locus, DNA barcode, Molecular identification

## Abstract

The polychaete genus *Laeonereis* (Annelida, Nereididae) occurs over a broad geographic range and extends nearly across the entire Atlantic coast of America, from the USA to Uruguay. Despite the research efforts to clarify its diversity and systematics, mostly by morphological and ecological evidence, there is still uncertainty, mainly concerning the species *Laeonereis culveri*, which constitutes an old and notorious case of taxonomic ambiguity. Here, we revised the molecular diversity and distribution of *Laeonereis* species based on a multi-locus approach, including DNA sequence analyses of partial segments of the cytochrome c oxidase subunit I (COI), 16S rRNA, and 28S rRNA genes. We examined *Laeonereis* specimens collected from 26 sites along the American Atlantic coast from Massachusetts (USA) to Mar del Plata (Argentina). Although no comprehensive morphological examination was performed between different populations, the COI barcodes revealed seven highly divergent MOTUs, with a mean K2P genetic distance of 16.9% (from 6.8% to 21.9%), which was confirmed through four clustering algorithms. All MOTUs were geographically segregated, except for MOTUs 6 and 7 from southeastern Brazil, which presented partially overlapping ranges between Rio de Janeiro and São Paulo coast. Sequence data obtained from 16S rRNA and 28S rRNA markers supported the same MOTU delimitation and geographic segregation as those of COI, providing further evidence for the existence of seven deeply divergent lineages within the genus. The extent of genetic divergence between MOTUs observed in our study fits comfortably within the range reported for species of polychaetes, including Nereididae, thus providing a strong indication that they might constitute separate species. These results may therefore pave the way for integrative taxonomic studies, aiming to clarify the taxonomic status of the *Laeonereis* MOTUs herein reported.

## Introduction

The genus *Laeonereis* Hartman, 1945 occurs over a broad geographic range and extends nearly across the entire western Atlantic Coast, from North Carolina (USA) to Canelones (Uruguay) (Jesús-Flores, Salazar-Gonzáles & Salazar-Vallejo, 2016; De León-González, Méndez & Navedo, 2018). A single species occurs from El Salvador to Costa Rica on the Pacific Coast of Central America, while another one was described occurring on the Gulf of California, Mexico (Jesús-Flores, Salazar-Gonzáles & Salazar-Vallejo, 2016; De León-González, Méndez & Navedo, 2018). The monospecificity of *Laeonereis* proposed by Pettibone (1971) has been questioned, resulting in the validation of six species that occur along the Atlantic and Pacific American coasts, namely *Laeonereis culveri* (Webster, 1879) (North America coast; Orensanz & Gianuca, 1974), *L. nota* (Treadwell, 1941) (Central America coast; Jesús-Flores, Salazar-Gonzáles & Salazar-Vallejo, 2016), *L. acuta* (Treadwell, 1923) (South America coast; Orensanz & Gianuca, 1974; Santos & Lana, 2001; Liñero-Arana & Díaz-Díaz, 2007; Pamplin, Almeida & Silva-Filho, 2007), *L. pandoensis* (Monro, 1937), *L. watsoni* De León-González, Méndez & Navedo, 2018 (Gulf of California; De León-González, Méndez & Navedo, 2018), and *L. brunnea* (Central America Pacific coast; Dean, 2001; Dean, Sibaja-Cordero & Cortés, 2012).

The uncertainties about the actual diversity of this genus come from scarcity of molecular data and difficult species identification (mainly by non-taxonomists), which is due to intraspecific morphological variability and subtle diagnostic characters (Jesús-Flores, Salazar-Gonzáles & Salazar-Vallejo, 2016; De León-González, Méndez & Navedo, 2018; Oliveira et al., 2010), besides a lack of updated information in reference biological and genetic databases, such as Zoobank, GenBank, and BOLD. In addition to the taxonomic issues addressed in several studies, the case of *Laeonereis* merits particular attention because of its relevance in biomonitoring. As a well-represented polychaete in estuarine environments from the western Atlantic Ocean (including the Gulf of Mexico and the Caribbean Sea), it has been used as a target species in population ecology and ecotoxicological studies, as well as in water and sediment quality monitoring surveys (Omena & Amaral, 2001; Rosa et al., 2005; Weis et al., 2017; Carricavur et al., 2018; Checon et al., 2018).

Estuaries constitute a prominent environment throughout the coastal region where invertebrate populations can be subject to some degree of confinement (Seixas, Paiva & Russo, 2018). However, this has not prevented many typical estuarine species from being considered cosmopolitan or at least widely distributed (Virgilio et al., 2009; Tomioka et al., 2016; Silva et al., 2017). Estuarine polychaete species have received special attention, mainly for their roles in communities and potential for biomonitoring surveys of benthic fauna (Winter, Devictor & Schweiger, 2013; Nygren, 2014; Carricavur et al., 2018). Therefore, to guarantee the success and accuracy of benthic surveys and allow their replication worldwide (Winter, Devictor & Schweiger, 2013), the correct species identification within these communities is fundamental. Moreover, estuarine *Laeonereis* species can occur in very high densities, which are stimulated by a small freshwater inflow from nearby streams. For example, *L. culveri* is one of the most common annelids in Alligator (Florida) and in the Mystic River estuary (Connecticut), where it occurs in aggregates, reaching up to 10 cm deep in fine sand and withstands variations in a salinity range of 0.5–30‰ (Mazurkiewicz, 1975). In the southwest coast of Brazil (São Paulo), high densities were found at the high intertidal areas of beaches and close to streams; in the Porto do Saco da Ribeira (Ubatuba), it reached more than 5,600 individuals/m^2^ and a biomass wet weight above 150 g/m², which constitutes a significant contribution to the area (Amaral, 1979).

Given the sustained difficulties faced by researchers throughout decades to elucidate *Laeonereis* taxonomy using morphology, and considering its relevance in biomonitoring, the present study aimed to apply a molecular approach to examine the diversity of *Laeonereis* populations collected along the western Atlantic Ocean and investigate their geographic distribution. For this purpose, we used multi-locus sequence data generated by two mitochondrial genes, namely the 5’ end of the cytochrome c oxidase subunit I (COI) and 16S rDNA (16S), and one nuclear locus, the 28S rDNA (28S). Molecular approaches have been successfully applied to contribute and elucidate the diversity of polychaetes, particularly over the last decade (Brasier et al., 2017; Lobo et al., 2016; Nygren et al., 2018; Langeneck et al., 2020; Teixeira et al., 2020). Here, we aimed to generate the basal molecular assessment of west Atlantic *Laeonereis* to pave the way for future integrative taxonomy studies on this genus.

## Materials and Methods

### Specimen sampling

**Figure 1:**
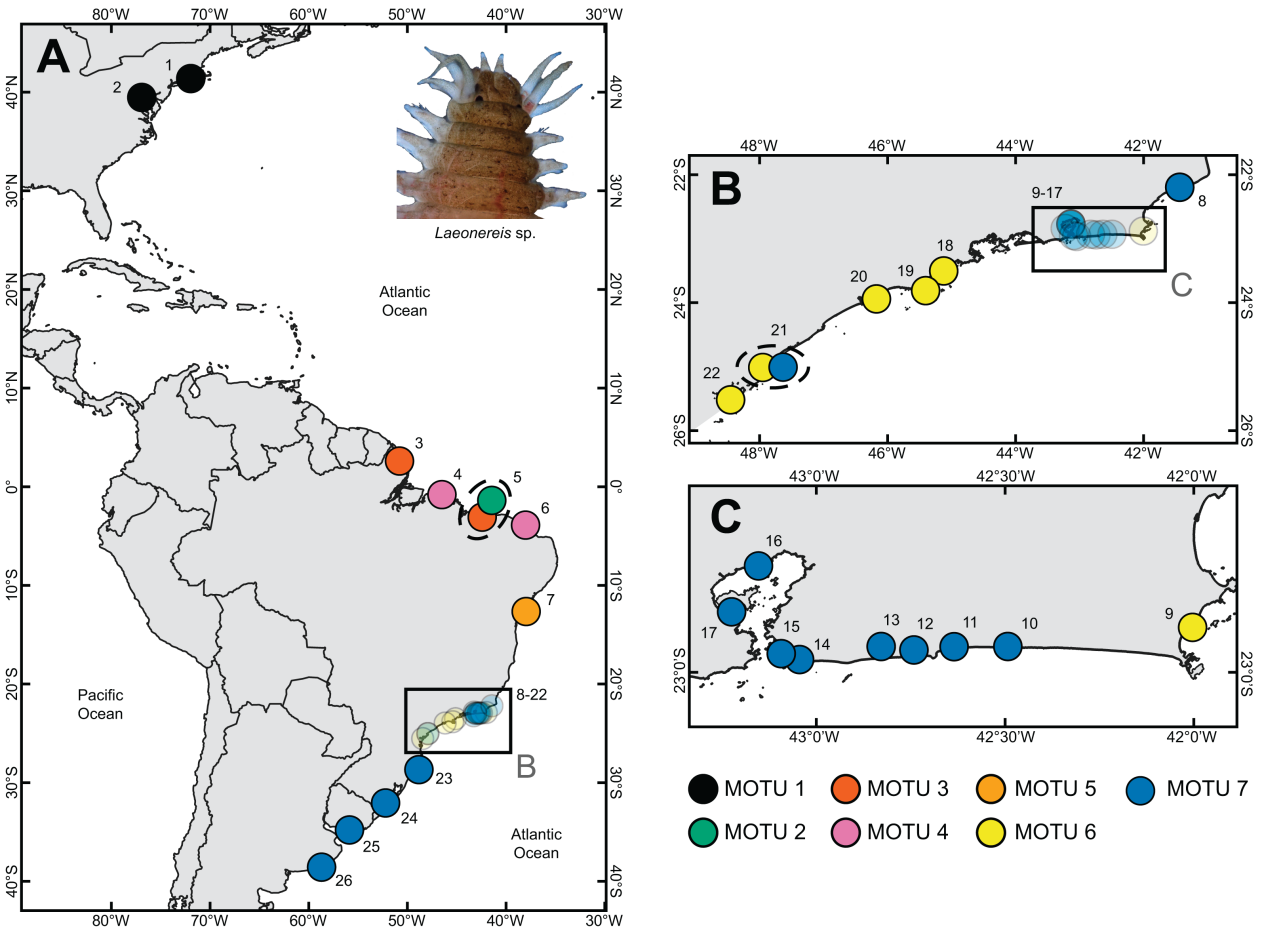
Sample sites where specimens of *Laeonereis* species were collected. (A) Map with the total extent of the sampling. (B and C). Approximate maps of samples from southeastern BRA. Dotted ellipse indicates samples from the same location. Sites: **1.** Connecticut, USA; **2.** Maryland, USA; **3.** Amapá, BRA; **4.** Pará, BRA; **5.** Piauí, BRA; **6.** Ceará, BRA; **7.** Bahia, BRA; **8.** Lagoa do Visgueiro, Rio de Janeiro, BRA; **9.** Ilha do Japonês, Rio de Janeiro, BRA; **10.** Lagoa de Saquarema, Rio de Janeiro, BRA; **11.** Lagoa de Jaconé, Rio de Janeiro, BRA; **12.** Lagoa de Guarapiná, Rio de Janeiro, BRA; **13.** Lagoa de Maricá, Rio de Janeiro, BRA; **14.** Lagoa de Itaipú, Rio de Janeiro, BRA; **15.** Lagoa de Piratininga, Rio de Janeiro, BRA; **16.** Praia da Coroa, Rio de Janeiro, BRA; **17.** Ilha do Fundão, Rio de Janeiro, BRA; **18.** Ubatuba, São Paulo, BRA; **19.** São Sebastião, São Paulo, BRA; **20.** Guarujá, São Paulo, BRA; **21.** Cananéia, São Paulo, BRA; **22.** Paraná, BRA; **23.** Santa Catarina, BRA; **24.** Rio Grande do Sul, BRA; **25.** Montevideo, URU; **26.** Mar del Plata, ARG.

Specimens of *Laeonereis* were collected from 26 localities between August 2016 and December 2017, two on the east coast of the United States (USA), 22 along the coast of Brazil (BRA: Amapá State [AP]; Bahia State [BA]; Ceará State [CE]; Paraná State [PR]; Piauí State [PI]; Rio de Janeiro State [RJ]; Rio Grande do Sul State [RS]; Santa Catarina State [SC]; São Paulo State [SP]), plus one in Uruguay (URU) and one in Argentina (ARG) (Fig. 1). To this end, soft-bottom samples were taken with shovels or sediment corers in the intertidal zone during syzygy tide. All specimens were identified morphologically and confirmed as belonging to the *Laeonereis* genus, according to a set of morphological characters available in the literature (Hartman, 1938; 1945; Pettibone, 1971; Orensanz & Gianuca, 1974; Dean, 2001; Santos & Lana, 2001; Liñero-Arana & Díaz-Díaz, 2007; Pamplin, Almeida & Silva-Filho, 2007; Oliveira et al., 2010; Dean, Sibaja-Cordero & Cortés, 2012; Jesús-Flores, Salazar-Gonzáles & Salazar-Vallejo, 2016), as follow: sub-pyriform prostomium; pair of frontal antennae; four pairs of frontal tentacular cirri; eversible pharynx with a pair of mandibles and tufts of soft papillae within proximal and distal rings; biramous parapodia with small ventral and dorsal cirri; and pygidium with a pair of cirri. Figure S1 presents photomicrographs and illustrations of the main characters used for the genus identification.

### DNA extraction, amplification, and sequencing

DNA was extracted from a small piece of tissue from each specimen, using the E.Z.N.A® Mollusc DNA Kit (Omega Bio-Tek, Norcross, GA, USA) and following the manufacturer’s protocol. The following molecular markers were used in this study: cytochrome c oxidase subunit I gene (COI), 16S ribosomal DNA gene (16S), and 28S ribosomal DNA gene (28S). Amplification reactions were performed in a MyCycler™ (Bio-Rad, Hercules, CA, USA) thermocycler using the following protocol: 2.5 μl of 10× PCR buffer, 1.5 ul (10 mM COI) or 2.5 ul (10 nM 16S/28S), 2.5 ul of 25 mM of MgCL_2_, 0.5 μl of 10uM dNTP mixture, 0.2 μl of DNA *Taq* polymerase (Thermo Scientific, Waltham, MA, USA). PCR conditions and primer sequences for each genetic marker are listed in Table S1. PCR products were verified on 1.5% agarose gel and cleaned up using ExoSap (Thermo Scientific, Waltham, MA, USA). Cleaned-up amplicons were sent to an external sequencing service for bidirectional sequencing (Macrogen, Spain and STAB Vida, Portugal).

Table 1 details the specimen collection and sampling locations. Table S2 lists the GenBank and BOLD accession numbers for all sequences retrieved from databases and the voucher number in the Polychaete Collection of the National Museum of Rio de Janeiro and the Zoological Museum of the University of Campinas (ZUEC-UNICAMP). All samples from the states of Connecticut (USA) and Amapá (Brazil) were used for DNA extraction. However, both populations are well represented among the lineages (MOTU) detected in this study; meanwhile, they also have representative specimens deposited in scientific collections. All sequences, trace files, and metadata were deposited in the BOLD system’s database within the dataset “**DS – LCSC: *Laeonereis culveri* species complex**” available at DOI: dx.doi.org/10.5883/DS-LCSC.

**Table 1:** List of species used in the present study, source of sequences and geographical coordinates.

Species	MOTU	Locality	Source	Latitude	Longitude
*Laeonereis culveri*	MOTU 1	Cheesepeak Bay, Maryland, USA	GenBank	38°88′71″N 38°88′99″N	76°52′64″W 76°53′09″W
*Laeonereis culveri*	MOTU 1	Stonington, Connecticut, USA	Present study	41°19’00.0″N	71°58’00.0″W
*Laeonereis* sp.	MOTU 2	Delta do Parnaíba, Parnaíba, PI, Brazil	Present study	2°51′45.09″S	41°39′57.70″W
*Laeonereis* sp.	MOTU 3	Praia do Goiabal, Calçoene, AP, Brazil	Present study	2°35′34.7″N	50°50′46.06″W
*Laeonereis* sp.	MOTU 3	Delta do Parnaíba, Parnaíba, PI, Brazil	Present study	2°51′45.09″S	41°39′57.70″W
*Laeonereis* sp.	MOTU 4	Praia de Ajuruteua, Bragança, PA, Brazil	Present study	0°50′19.16″S	46°35′55.15″W
*Laeonereis* sp.	MOTU 4	Rio Pacoti, Fortaleza, CE, Brazil	Present study	3°49′51.53″S	38°25′11.03″W
*Laeonereis* sp.	MOTU 5	Praia do Forte, Mata de São João, BA, Brazil	Present study	12°34′59.24″S	38° 0′52.94″W
*Laeonereis* sp.	MOTU 6	Ilha do Japonês, Cabo Frio, RJ, Brazil	Present study	22°52′50.80″S	42° 0′10.80″W
*Laeonereis* sp.	MOTU 6	Saco da Ribeira, Ubatuba, SP, Brasil	Present study	23°30′16.72″S	45° 7′19.99″W
*Laeonereis* sp.	MOTU 6	Baía do Araçá, São Sebastião, SP, Brasil	Present study	23°48′45.36″S	45°24′18.33″W
*Laeonereis* sp.	MOTU 6	Praia do Perequê, Guarujá, SP, Brasil	Present study	23°56′30.51″S	46°10′25.27″W
*Laeonereis* sp.	MOTU 6	Canal de Cananéia, Cananéia, SP, Brasil	Present study	25° 0′49.36″S	47°55′35.69″W
*Laeonereis* sp.	MOTU 6	Ilha da Cotinga, Paranaguá, PR, Brasil	Present study	25°31′9.74″S	48°27′11.74″W
*Laeonereis* sp.	MOTU 7	Lagoa de Guarapiná, Maricá, RJ, Brazil	Genbank	22°56′26.21″S	42°44′28.87″W
*Laeonereis* sp.	MOTU 7	Baía de Guanabara, RJ, Brazil	Present Study	22°71′84.53″S	43°16′46.47″W
*Laeonereis* sp.	MOTU 7	Ilha do Fundão, Baía de Guanabara, RJ, Brazil	GenBank	22°50′22.44″S	43°13′28.19″W
*Laeonereis* sp.	MOTU 7	Lagoa de Guarapiná, Maricá, RJ, Brazil	GenBank	22°56′26.21″S	42°44′28.87″W
*Laeonereis* sp.	MOTU 7	Lagoa de Itaipú, Niterói, RJ, Brazil	GenBank	22°57′57.86″S	43° 2′39.69″W
*Laeonereis* sp.	MOTU 7	Lagoa de Jaconé, Saquarema, RJ, Brazil	GenBank	22°55′55.13″S	42°38′5.86″W
*Laeonereis* sp.	MOTU 7	Lagoa de Maricá, Maricá, RJ, Brazil	GenBank	22°55′53.34″S	42°49′41.59″W
*Laeonereis* sp.	MOTU 7	Lagoa de Saquarema, Saquarema, RJ, Brazil	GenBank	22°55′53.12″S	42°29′33.01″W
*Laeonereis* sp.	MOTU 7	Lagoa de Piratininga, Niterói, RJ, Brazil	GenBank	22°57′1.62″S	43° 5′34.71″W
*Laeonereis* sp.	MOTU 7	Lagoa do Visgueiro, Quissamã, RJ, Brazil	GenBank	22°11′47.36″S	41°25′59.84″W
*Laeonereis* sp.	MOTU 7	Canal de Cananéia, Cananéia, SP, Brasil	Present study	25° 0′49.36″S	47°55′35.69″W
*Laeonereis* sp.	MOTU 7	Lagoa do Imaruí, Laguna, SC, Brasil	Present study	28°27′43.2″S	48°47′50.7″W
*Laeonereis* sp.	MOTU 7	Saco do Justino, Rio Grande, RS, Brasil	Present study	32° 5′5.68″S	52°13′7.45″W
*Laeonereis* sp.	MOTU 7	Canelones, Montevideo, Uruguai	Present study	34°47′41.41″S	55°52′32.75″W
*Laeonereis* sp.	MOTU 7	Rio Quequén, Mar del Plata, Argentina	Present study	38°33′51.60″S	58°42′41.36″W
*Ceratocephale* cf. *loveni*	OUTGROUP	Nova Scotia, Canada	GenBank	–	–
*Micronereis nanaimoensis*	OUTGROUP	British Columbia, Canada	GenBank	53°40′26.4"N	132°22′37.2"W
*Allita succinea*	OUTGROUP	–	GenBank	–	–
*Allita succinea*	OUTGROUP	Maryland, USA	GenBank	38°88′06"N	76°54′02"W
*Allita succinea*	OUTGROUP	Mar Chiquita, Mar del Plata, Argentina	Present study	37°44′27.47"S	57°25′13.97"W

### Sequence data treatment

The forward and reverse trace files of the three DNA markers were checked and edited in MEGA 7.0 software (Kumar, Stecher & Tamura, 2016). COI and 16S sequences were aligned by Clustal W software (Thompson, Higgins & Gibson, 1994) and 28S sequences by Muscle software (Edgar, 2004). A total of 81, 58, and 45 original sequences of COI (589 bp), 16S (442 bp), and 28S (339 bp) were considered representatives of the genus *Laeonereis*, respectively. The 5’ and 3’ ends were pruned according to the quality, and all sequences were “normalized” to the minimum-length sequence. For the 28S marker, the selected primer amplified an 800 bp fragment, which included the hypervariable region in the fragment (Nygren et al., 2018). Therefore, the hypervariable zone was excluded for phylogenetic analyses and genetic distance estimation, resulting in a 339 bp fragment. Given the presence of gaps and nucleotide positions with poor 28S alignment quality, the online tool Gblocks 0.91b (Castresana, 2000) (http://molevol.cmima.csic.es/castresana/Gblocks.html) was used to optimize alignment blocks for phylogenetic analyses. After optimization, nearly 15% of the bases were removed from original sequences, resulting in a fragment of 293 bp.

### Phylogenetic reconstruction

The phylogenetic relationships between populations were reconstructed through Bayesian inference (BI) and maximum likelihood (ML). The alignments of each locus were analysed individually. The best-fit substitution models for each locus were determined using the best-fit model tool in MEGA 7.0 (for ML) and jModelTest (for BI), which are based on the Bayesian Information Criterion (BIC) (Guindon & Gascuel, 2003; Darriba et al., 2012). For COI, specific models were determined for each position of the codon in the BI analysis. In other words, the Hasegawa-Kishino-Yano model with gamma distribution and the invariant sites (HKY+G+I) model were selected for the first two codon positions, while the General Time Reversible (GTR+G+I) model for the third codon position. For the ML analysis with COI, the GTR+G+I model was used. For 16S and 28S, the GTR+G+I and HKY models were applied for both methods, respectively.

Bayesian inference was performed in MrBayes v.3.1.2 (Ronquist & Huelsenbeck, 2003) with two parallel runs, using 10 million generations and sampling parameters every 500 generations. One-quarter of the trees were discarded as burn-in. The average standard deviation of split frequencies was confirmed for each analysis, with values below 0.02, indicating tree convergence (Ronquist & Huelsenbeck, 2003). The ML phylogenies were estimated in MEGA 7.0 software, using the NNI heuristic method and branch supports estimated with 1000 bootstraps replications. The resulting trees were analysed in the software FigTree 1.4.3 (http://tree.bio.ed.ac.uk/software/figtree/) to interpret and confirm branch supports and appropriate clade bifurcation, according to the model used. When low support or inadequate clade bifurcation were identified, a new run with other models was performed. The final versions of the trees were edited in Adobe Illustrator CC software (https://adobe.com/products/illustrator).

Two COI sequences of *Alitta succinea* (Leuckart, 1847) from Argentina were produced following the above protocol and used as an outgroup. In addition, four sequences of *A. succinea* (two COI and two 16S), one of *Micronereis nananimoensis* Berkeley & Berkeley, 1953 (COI), one of *Ceratocephale* cf. *loveni* Malmgren, 1867 (16S), and one of *Nereis succinea* (*Alitta succinea*) (28S) were retrieved from GenBank and BOLD to be used as an outgroup in the phylogeny of each locus (Table 1).

Raw data (alignment and phylogenetic trees) were deposited and are publicly available at Figshare (Dataset - https://doi.org/10.6084/m9.figshare.12733148.v1; Trees - https://doi.org/10.6084/m9.figshare.12733043.v1).

### Delimitation of Molecular Operational Taxonomic Units (MOTU)

The molecular dataset was subjected to four MOTU delimitation methods as previously described in Vieira et al. (2019), Desiderato et al. (2019), and Teixeira et al. (2020); two distance-based (BIN and ABGD) and two phylogeny-based (GMYC and bPTP). These methods were applied to all studied loci except the BIN method, which is implemented within the BOLD system (Ratnasingham & Hebert, 2013) and applies only to COI data. This approach clusters barcode sequences algorithmically to calculate MOTUs that show high concordance to species (Ratnasingham & Hebert, 2013). The Automatic Barcode Gap Discovery (ABGD) species delimitation tool was performed on a web interface and applied with default settings, using the genetic distance matrix of Kimura-2-Parameters (K2P). This tool allows the sorting of DNA sequences into hypothetical species, based on barcode gap detection (Puillandre et al., 2012).

The Generalized Mixed Yule Coalescent method (GYMC; Fujisawa & Barraclough, 2013) is based on the examination of the branching patterns of an ultrametric tree and recognition of their transitions attributable to speciation (one lineage per species) to those that can be attributed to the interspecies coalescence process (multiple lineages per species). The single-threshold variant of this method was applied (Pons et al., 2006). The Bayesian ultrametric tree was generated in BEAST 2.4.6 (Bouckaert et al., 2014) with the appropriate best model (based on AIC criteria, GTR+I) and four independent series of 50,000,000 Monte Carlo Markov Chain (MCMC) generations, sampled every 5,000 generations. Quality control analysis was performed in the Tracer 1.6 software (Rambaut et al., 2014) evaluating the ESS (Effective Sample Size) (ESSs > 200 for all parameters) and parameter estimation convergence. A consensus tree was obtained using TreeAnnotator v.2.4.6 (Bouckaert et al., 2014) and visualized in FigTree 1.4.3.

The Poisson Tree Processes (bPTP) method incorporates the number of substitutions in the model of speciation and assumes that the probability that a substitution gives rise to a speciation event follows a Poisson distribution. The branch lengths of the input tree are supposed to be generated by two independent classes of the Poisson process, one corresponding to speciation and the other to coalescence (Zhang et al., 2013). In contrast to GMYC, bPTP accepts non-ultrametric trees; thus, the previously estimated ML tree was used. Both GMYC and bPTP analyses were performed on a web interface (https://species.h-its.org/).

### Genetic diversity and structure

The genetic distances between and within MOTUs were calculated in the MEGA 7.0, using the K2P model. To evaluate the relationship between haplotypes and their geographical distribution, haplotype networks were built using the TCS method (Clement, Snell & Wlaker, 2001) as implemented in PopART software (Leigh & Bryant, 2015). For this purpose, the data from the six different sites in Rio de Janeiro, as well as the four in São Paulo, were grouped in two locations, RJ and SP, respectively. Indices of genetic diversity, namely haplotype diversity (h) and nucleotide diversity (π), were estimated for each MOTU based on COI data and using DNASP 5.10 (Table S3) (Librado & Rozas, 2009).

## Results

### Phylogenetic inference and MOTU delimitation

**Figure 2:**
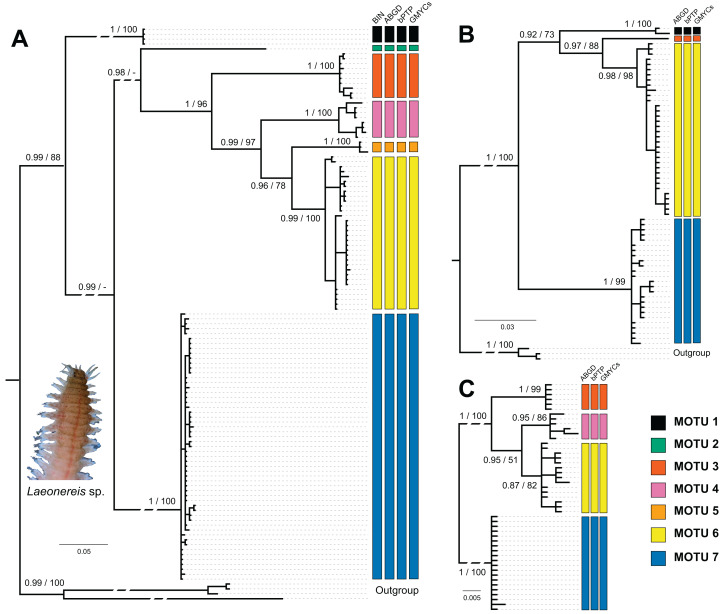
Bayesian phylogenetic trees for *Laeonereis* species based on COI (A), 16S (B) and 28S (C). Node values represents posterior probability (Bayesian inference) and bootstrap support (maximum likelihood). Nodes with a dash were not recovered in maximum likelihood analysis. Vertical coloured bars represent the results of molecular species delimitation methods, BIN (only for COI), ABGD, bPTP and GYMC single threshold.

Sampling sites and geographic sources of sequences retrieved from databases are illustrated and detailed in Fig. 1, in which the distribution of MOTUs along the North and South America coasts can be observed (Fig. 1). Phylogenetic inference and the four automated delimitation methods are congruent for all molecular markers (except the BIN method which is available only for COI). The phylogenetic tree of COI recovered seven reciprocally monophyletic groups, with branch support above 0.95 (BI) and 90 (ML) (Fig. 2A). MOTU 1 included specimens from the USA only; MOTUs 2 (BRA – PI), 3 (BRA – AP and PI), 4 (BRA – PA and CE), and 5 (BRA – BA) were exclusive to the North and Northeast regions of Brazil; and MOTUs 6 (BRA – RJ, SP and PR) and 7 (BRA – RJ, SP, SC, and RS; URU; and ARG) were exclusive to the Southeastern and Southern regions of Brazil; with the latter also being found in Uruguay and Argentina. MOTU 2 contained only one sequence from a specimen caught at the same site as that of MOTU 3 (PI). All different delimitation methods applied to COI resulted in the same partitioning, also separating *L. culveri* into seven MOTUs, except for the BIN method in which MOTU 6 was split into three different groups, resulting in nine MOTUs.

The number of specimens and localities represented in 16S (Fig. 2B) and 28S (Fig. 2C) databases are lower than those in COI, and so were the numbers of clades and MOTUs. The clade recovery in the 16S and 28S trees matches that found in the COI tree, and in all cases, node support was higher than 0.9 (BI) or 90 (ML). All delimitation methods corroborated the phylogenetic results for both markers. The 16S sequences represented MOTUs 1, 3, 6, and 7, while the 28S sequences represented MOTUs 3, 4, 6, and 7.

### Genetic distance and diversity

The overall mean genetic distance (K2P) within the 113 *Laeonereis* sequences of COI was 11.6%. Table 2 shows the genetic distances for each marker, within and among MOTUs. All MOTUs showed low within-MOTU genetic variability (<1%) but high between-MOTU genetic distance. For the COI dataset, the mean genetic distances within and among MOTUs were 0.7% (0.0–1.7%) and 16.9% (6.8–21.9%), respectively. In the 16S dataset, these values were, respectively, 0.3% (0.0–0.3%) and 9.2% (4.5–11.3%), while for the 28S they were 0.7% (0.0–1.2%) and 5.4% (1.4–7.5%). Regarding genetic diversity, MOTUs 1 and 2 were monomorphic, MOTU 6 (RJ, SP, PR) presented the highest nucleotide diversity (0.02183) and segregating sites (105), while MOTU 7 (RJ, SP, SC, RS, URU, and ARG) had the highest haplotype diversity (0.869) (Table S3).

**Table 2:** Inter and Intraspecific (in bold) mean distances (K2P) with 1000 bootstrap and standard deviation for COI, 16S and 28S, respectively for each MOTU.

	1	2	3	4	5	6	7
MOTU 1	**0.0 ± 0.0** **0.5 ± 0.3** **NA**						
MOTU 2	19.6 ± 2.0 NA NA	**NA** **NA** **NA**					
MOTU 3	18.5 ± 1.9 9.0 ± 1.4 NA	20.0 ± 2.0 NA NA	**0.4 ± 0.1** **NA** **0.0 ± 0.0**				
MOTU 4	20.7 ± 2.1 NA NA	18.8 ± 2.0 NA NA	15.5 ± 1.6 NA 4.3 ± 1.2	**1.7 ± 0.4** **NA** **0.4 ± 0.2**			
MOTU 5	20.7 ± 2.1 NA NA	16.5 ± 1.8 NA NA	15.5 ± 1.8 NA NA	10.8 ± 1.4 NA NA	**0.3 ± 0.2** **NA** **NA**		
MOTU 6	18.8 ± 1.9 7.9 ± 1.0 NA	17.0 ± 1.9 NA NA	14.9 ± 1.6 4.8 ± 1.0 4.0 ± 1.1	10.8 ± 1.4 NA 1.8 ± 0.7	7.7 ± 1.1 NA NA	**1.3 ± 0.3** **0.3 ± 0.1** **0.3 ± 0.2**	
MOTU 7	17.5 ± 1.8 10.8 ± 1.6 NA	15.1 ± 1.6 NA NA	18.5 ± 1.8 9.7 ± 1.6 7.1 ± 2.7	19.6 ± 1.9 NA 5.6 ± 1.3	17.5 ± 1.8 NA NA	17.6 ± 1.8 9.4 ± 1.5 6.2 ± 1.3	**1.2 ± 0.1** **0.3 ± 0.2** **0.0 ± 0.0**

### Haplotype network

**Figure 3:**
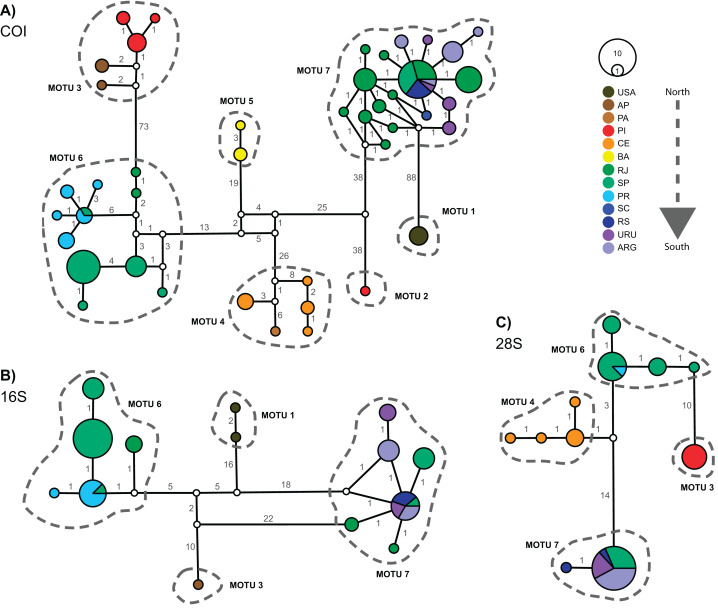
Haplotype networks for *Laeonereis* species based on TCS method for all three markers COI (A), 16S (B) and 28S (C). Each haplotype is represented by a circle and the specimen number of each haplotype is represented by its size. Colors are indicative of the geographical specimen’s origin and number over lines indicate the number of mutations. **ARG** = Mar del Plata, Argentina; **AP** = Amapá, Brazil; **BA** = Bahia, Brazil; **CE** = Ceará, Brazil; **PA** = Pará, Brazil; **PI** = Piauí, Brazil; **PR** = Paraná, Brazil; **RJ** = Rio de Janeiro, Brazil; **RS** = Rio Grande de Sul, Brazil; **SC** = Santa Catarina, Brazil; **URU** = Uruguay; **USA** = United States.

Figure 3 displays the haplotype networks for all three markers. The COI haplotype network (Fig. 3A) showed 43 haplotypes distributed among 114 specimens. None of the MOTUs had a central position in the network. The highest number of mutations observed between two linked MOTUs was 88 (MOTU 1 – USA × MOTU 7 – Argentina, Uruguay, and South Brazil) and the lowest was 38 (MOTU 5 – BA, Northeast Brazil × MOTU 6 – PR, SP, and RJ, South and Southeastern Brazil). Besides, only two MOTUs occur in the same locations: in MOTU 6 a haplotype occured in SP and PR, while MOTU 7 occured in RJ, SP, RS, URU, and ARG (Fig. 3A). The 16S and 28S haplotype networks (Figs. 3B and 3C) corroborated the COI results. As only subsets of localities were represented, 16S and 28S presented only four MOTUs each (as mentioned in the “Phylogenetic Inference and MOTU Delimitation” section). Considering the MOTUs 6 and 7 (represented in all markers), the localities sharing haplotypes in COI were the same as observed in 16S and 28S (except RJ).

## Discussion

Molecular approaches, such as DNA barcoding, have recently emerged as particularly effective tools for species identification, enabling non-taxonomists to assess diversity in a variety of scientific fields, from environmental sciences to forensics. Some authors even argue that these methods will become an essential component of taxonomic revisions and biodiversity surveys (Stein et al., 2014; DeSalle & Goldstein, 2019). During the last decade, COI barcodes have been used as reliable marker to provide diagnostic characters for species delimitation, elucidating species boundaries within several taxa (Delić et al., 2017; Teixeira et al., 2020). The molecular analysis of the genus *Laeonereis* performed herein confirmed the existence of a high genetic diversity within the studied populations and revealed at least seven deeply divergent lineages, six of which are distributed along the Atlantic coast of South America. These lineages may include several of the previously proposed species based on morphological characters (i.e., *L. acuta*, *L. culveri*, *L. nota*, *L. pandoensis*; Orensanz & Gianuca, 1974; Santos & Lana, 2001; Jesús-Flores, Salazar-Gonzáles & Salazar-Vallejo, 2016). However, before being inferred, the concrete matches with specific MOTUs reported here require further studies for confirmation.

The genetic distances recorded among *Laeonereis* MOTUs (COI: 6.8–21.9%) fit perfectly within the range reported for congeneric distances in several other studies with polychaetes. For example, comprehensive studies on the polychaete fauna of the Arctic (Carr et al., 2011) and north-eastern Atlantic (Lobo et al., 2016) reported mean COI congeneric distances (K2P) of 16.5% and 24.0%, respectively, vs the 16.9% observed here. Our findings are also consistent with pairwise comparisons among closely-related species observed both within Nereididae (e.g., Glasby, Wei & Gibb, 2013; Paiva et al., 2019) and in other polychaete families, such as Amphinomidae (Barroso et al., 2010), Capitellidae (Silva et al., 2017), Onuphidae (Rodrigues et al., 2009; Seixas et al., 2020), Sabellidae (Capa & Murray, 2015), Phyllodocidae (Ravara et al., 2017), and Syllidae (Álvarez-Campos, Giribet & Riesgo, 2017).

The high substitution rates of mitochondrial genes found here, for COI and 16S, as well as their ability to discriminate species, have already been widely reported in the literature (e.g., Brasier et al., 2017; Hupało et al., 2019; Tosuji et al., 2019; Vieira et al., 2019). On the other hand, nuclear ribosomal genes had low mutation rates and are best suited to reconstruct deep phylogenies. Thus, both COI and 16S genetic distances found herein were congruent, although the 16S sequence data had not been produced for all MOTUs. The observed distances for 28S were unexpected and, despite lower than those of the other markers, they exhibited higher distances (1.4–7.5%) than what is usually reported for other polychaetes (Tosuji et al., 2019; Teixeira et al., 2020). Thus, in our study, the 28S interspecific genetic distances for this group corroborated the species delimitation found for the COI and 16S.

Considering that *Laeonereis* species occurs predominantly in transitions between freshwater and marine ecosystems (e.g., estuarine, bays, coastal lagoons, and river mouths), such conditions could hypothetically promote evolutionary segregation, particularly over a broad distribution range. Moreover, the fact that *L. culveri* has benthic larvae that hatch from eggs fertilized inside the mucoid tube of females (Mazurkiewicz, 1975), could also contribute to limiting connectivity among populations. Although the larvae have a certain vertical locomotion/swimming capacity associated with food scavenging (Klesch, 1970, Mazurkiewicz, 1975), the time spent in the water column is short compared to species with fully planktonic larvae. All together, these features point to a comparatively low dispersal potential, as assumed by De León-González, Méndez & Navedo (2018). In these cases, strategies of larval retention or exportation, such as timing and location of egg or larval release, have great relevance for dispersal capacity (Bilton, Paula & Bishop, 2002).

The distribution of MOTUs within the genus *Laeonereis* is well marked by differences in oceanographic conditions. MOTU 1 is restricted to the east coast of the USA and is separated from the other MOTUs by thousands of kilometres. The MOTUs 2, 3, and 4 are associated with the northern Brazilian coast, which has a substantial contribution from river waters and terrestrial sediments (Dominguez, 2009). Hence, the salinity level of estuarine waters in the region is very close to that of freshwater. This region is also under the influence of the North Brazilian Current, which flows towards the Caribbean Sea and is marked by higher water temperatures. MOTU 5 is found in a similar region, but with slightly lower freshwater input, water temperature, and productivity. This region is influenced by the Brazilian current flowing predominantly southward. Finally, MOTUs 6 and 7 were sampled in a region influenced by upwelling events due to a mass of cold water (i.e., South Atlantic Central Water), decreasing water temperature and increasing productivity (Coelho-Souza et al., 2012; Mazzini & Barth, 2013).

To a certain extent, the geographic arrangement of the found MOTUs can be associated with the biogeographic divisions proposed by Spalding et al. (2007). Thus, MOTU 1 is restricted to the Warm Temperate Northwest Atlantic Province; MOTUs 2, 3, and 4 to the North Brazil Shelf Province; MOTU 5 to the Tropical Southwestern Atlantic Province; and MOTUs 6 and 7 to the Warm Temperate Southwestern Atlantic Province. Furthermore, phylogeographic and population genetic studies on other marine invertebrates have also matched these biogeographic divisions (corals – Peluso et al., 2018; crustaceans – Mattos, Seixas & Paiva, 2019), suggesting dominant phylogeographic discontinuities in the region pervasive across multiple and diverse taxa.

The deep genetic breaks observed among *Laeonereis* lineages can be explained by either dispersal followed by vicariance events or vicariance followed by dispersal events. The origin of the genus cannot be inferred from the results of this work; however, given its distribution, two scenarios are plausible: (1) origin in the North Atlantic / Caribbean followed by dispersal to South America, or (2) origin in South America followed by dispersal to North America. Both explain the initial separation of MOTU 1 from the South American MOTUs (2 to 7). Since its establishment, freshwater discharge from the Amazon River to the Atlantic Ocean (~7 mya; Hoorn et al., 2010) has been responsible for speciation between the Caribbean and Brazilian populations (Sales et al., 2017; Dias et al., 2019). Considering the South American lineages, the first splitting might have occurred between south and north populations of South America (MOTU 7 × MOTUs 2–6), perhaps ~23° S latitude, where there is currently a biogeographic break marked by a transitional area of marine fauna (Floeter & Soares-Gomes, 1999). Biogeographical processes can be chronologically categorised once the centre of origin of the *Laeonereis* genus was known. As the tropical regions are considered biodiversity hotspots (Bowen et al., 2013), we can suggest that the distribution limits of the ancestral lineage (MOTUs 2 to 7) might have expanded to the southern limits during favourable (interglacial) periods and subsequently retracted in unfavourable periods (glaciations).

Pleistocene glaciations caused several episodes of sea-level fluctuation, which were more severe in polar and temperate regions, such as in southern South America. For example, the Great Patagonian Glaciation between 1.78–0.78 mya resulted in sea level decreasing, higher salinity levels, and changes in sea currents (Ehlers & Gibbard, 2007). At the end of these glacial periods, postglacial marine transgression occurred, and sea levels gradually rose again. These glaciation episodes could have caused the extinction of many southern populations, which might have been restricted to a few regions (refuges). On the other hand, such glaciation events may have induced the establishment of ancestral populations in northern regions, given the more stable environmental conditions, such as temperature, salinity, and habitat availability (Ehlers & Gibbard, 2007; Tomazelli, Dillenburg & Villwock, 2017).

According to a review on the evolutionary molecular divergence for several groups of marine invertebrate species, polychaetes exhibit a K2P divergence rate ranging from 3.5 to 4.7% MY^−1^ (Loeza-Quintana et al., 2019). Herein, the K2P distances between MOTUs ranged from 7.7 (MOTU 5 × 6) to 20.7% (MOTU 1 × 4 and 1 × 5). Thus, it is possible to infer that a probable divergence between *Laeonereis* species from North and South America lies within a time gap from a few to a dozen million years. Despite the high uncertainty of this estimate, it might allow us to insert the divergence of species of the *Laeonereis* complex into the timescale of the relevant biogeographic events cited above.

Although the Caribbean Sea was not sampled and northern Brazilian coasts under-explored in this study, the occurrence of *Laeonereis* species is well established in those areas. Therefore, either the nearby MOTUs (2, 3, and 4) or other yet undescribed species of the *Laeonereis* complex may also be present in these regions. Accordingly, the diversification of these lineages may have occurred in the Caribbean, followed by dispersal to the northern coasts of Brazil after speciation, or vice-versa. However, to reveal the complete evolutionary history of this genus, the Caribbean, Gulf of Mexico, and Pacific regions need to be investigated.

Although geographically close, MOTUs 6 and 7 showed divergence from one another. This may have occurred by population dispersal from the northeast to southeast, followed by gene flow interruption. It also explains the high genetic distance between them, whose mitochondrial and nuclear lineages are completely sorted. Thus, these MOTUs could have diverged in the distant past and currently occur in the same region due to post-speciation range expansion.

## Conclusions

The most recent papers on Laeonereis species diversity have demonstrated the unlikelihood of a cosmopolitan *L. culveri*, recovering five species, and describing a new one, *L. watsoni* (Jesús-Flores, Salazar-Gonzáles & Salazar-Vallejo, 2016; De León-González, Méndez & Navedo, 2018; as summarised in WoRMS). The authors of these studies revealed a set of morphological characters capable of separating species; however, in their own words, these characters are subtle and, to some extent, difficult to be observed, which might lead to misidentification by non-taxonomists and students.

In this sense, to place our finding in a global context, we reported seven distinct lineages of *Laeonereis* (putative species) for the American Atlantic coast. The molecular diversity found for *Laeonereis* was high and suggests hidden diversity with at least two additional undescribed species. A comprehensive morphological analysis of each MOTU is still needed to reach robust conclusions on *Laeonereis* diversity, including a comparative analysis with the types of valid species. Our findings expand the range of the genus Laeonereis, from Connecticut—USA to Mar del Plata—Argentina.

The molecular diversity here brought to light has raised new questions about *Laeonereis* diversity and distribution: does each MOTU revealed herein constitute a single species? If so, how many of them can be matched with morphologically-validated species and how many are new? Could MOTUs 6 and 7 correspond to *L. acuta* and *L. pandoensis* species, respectively? Lastly, could the MOTUs detected in the northern region of Brazil match *L. nota*? At this moment, we are not able to confidently answer these questions without a comprehensive morphological examination. However, our molecular dataset substantially contributes to further studies in this group, paving the way for integrative approaches to clarify the taxonomy of the genus.

## Supplemental Information

10.7717/peerj.11364/supp-1 Supplemental Information 1 Primer sequences, PCR cycle conditions and primer references.

10.7717/peerj.11364/supp-2 Supplemental Information 2 List of species used in the present study, with remarks on the sampling locality, GenBank accession number and BOLD code for each marker, as well as reference list. Voucher numbers: ZUEC-Poly are from Zoological Museum of University of Campinas (UNICAMP) and MNRJP from Nacional Museum of Rio de Janeiro; * = specimens were used entirely for DNA extraction.

10.7717/peerj.11364/supp-3 Supplemental Information 3 Indices of genetic diversity estimated, based on COI for each MOTU. Number of sequences (n); number of variables sites (S); number of haplotypes (h); haplotype diversity (Hd); nucleotide diversity (π).

10.7717/peerj.11364/supp-4 Supplemental Information 4 *Laeonereis sp*. (MOTU 3): morphological characters used for identification (genus level). **A-B:** Anterior region with details of antenae (**a**), palps (**p**) two pair of eyes (**e**) and everted pharinx with soft papilae (**pa**; **arrows**). **C-D:** Everted pharinx ilustration, with details of the papilae (**pa**) and mandibles (**m**). **E:** Fifth setigers parapodia. **F-G:** Pigidium. **dc:** dorsal cirri; **L:** lingule; **pc:** pigidial cirri; **vc:** ventral cirri; **white arrows:** pigidium rim.

## Additional Information and Declarations

### DNA Deposition

The following information was supplied regarding the deposition of DNA sequences: The sequences are available at BOLD: LCCS083-20, LCCS085-20, LCCS073-20, LCCS074-20, LCCS075-20, LCCS037-19, LCCS038-19, LCCS039-19, LCCS040-19, LCCS041-19, LCCS042-19, LCCS043-19, LCCS044-19, LCCS079-20, LCCS045-19, LCCS046-19, LCCS047-19, LCCS048-19, LCCS049-19, LCCS050-19, LCCS051-19, LCCS076-20, LCCS077-20, LCCS078-20, LCCS080-20, LCCS081-20, LCCS084-20, LCCS086-20, LCCS087-20, LCCS088-20, LCCS001-19, LCCS002-19, LCCS003-19, LCCS005-19, LCCS006-19, LCCS095-20, LCCS052-19, LCCS004-19, LCCS007-19, LCCS008-19, LCCS009-19, LCCS010-19, LCCS011-19, LCCS012-19, LCCS013-19, LCCS014-19, LCCS015-19, LCCS016-19, LCCS017-19, LCCS018-19, LCCS019-19, LCCS020-19, LCCS021-19, LCCS022-19, LCCS023-19, LCCS024-19, LCCS025-19, LCCS026-19, LCCS027-19, LCCS028-19, LCCS029-19, LCCS030-19, LCCS031-19, LCCS032-19, LCCS033-19, LCCS034-19, LCCS035-19, LCCS036-19, LCCS082-20, LCCS053-19, LCCS054-19, LCCS055-19, LCCS056-19, LCCS057-19, LCCS058-19, LCCS059-19, LCCS060-19, LCCS061-19, LCCS062-19, LCCS063-19, LCCS064-19, LCCS065-19, LCCS066-19, LCCS068-19, LCCS069-19, LCCS070-19, LCCS071-19, LCCS028-19, LCCS029-19, LCCS030-19, LCCS031-19, LCCS032-19, LCCS033-19, LCCS034-19, LCCS035-19, LCCS036-19, LCCS082-20, LCCS053-19, LCCS054-19, LCCS055-19, LCCS056-19, LCCS057-19, LCCS058-19, LCCS059-19, LCCS060-19, LCCS061-19, LCCS062-19, LCCS063-19, LCCS064-19, LCCS065-19, LCCS066-19, LCCS068-19, LCCS069-19, LCCS070-19, LCCS071-19, LCCS028-19, LCCS029-19, LCCS030-19, LCCS031-19, LCCS032-19, LCCS033-19, LCCS034-19, LCCS035-19, LCCS036-19, LCCS082-20, LCCS053-19, LCCS054-19, LCCS055-19, LCCS056-19, LCCS057-19, LCCS058-19, LCCS059-19, LCCS060-19, LCCS061-19, LCCS062-19, LCCS063-19, LCCS064-19, LCCS065-19, LCCS066-19, LCCS068-19, LCCS069-19, LCCS070-19, LCCS071-19, LCCS072-19.

### Data Availability

The following information was supplied regarding data availability: Sampieri, Bruno (2020): Alignment dataset. figshare. Dataset. DOI 10.6084/m9.figshare.12733148.v1. Sampieri, Bruno (2020): Bayesian Trees COI / 16S / 28S. figshare. Figure. DOI 10.6084/m9.figshare.12733043.v1.
