# Supplementary material for: Molecular diversity within the genus Laeonereis (Annelida, Nereididae) along the west Atlantic coast: paving the way for integrative taxonomy
Source: PeerJ. 2021 May 27;9:e11364. doi: 10.7717/peerj.11364 (PMC8164838; doi:10.7717/peerj.11364)
Supplement: Supplemental Information 1 [file peerj-09-11364-s001.docx]

**Table S1**

| Marker | Primer | Sequence 5’-3’ | Lenght | PCR cycle | Reference |
| --- | --- | --- | --- | --- | --- |
| COI | PolyLCO | (F) GAYTATWTTCAACAAATCATAAAGATATTGG | 658 bp | 1)94 °C (1 min); 2) 5 cycles: 94 °C (40 s), 45 °C (40s), 72 °C (1 min); 3) 35 cycles: 94 °C (40 s), 51 °C (40 s), 72 °C (1 min); 4)72 °C (5 min). | Carr et al., 2011 |
|  | PolyHCO | (R) TAMACTTCWGGGTGACCAAARAATCA |  |  |  |
| 16S | 16SAr_F | (F) CGCCTGTTTATCAAAAACAT | 440 bp | 1)94°C (2 min); 2)35 cycles: 94°C (30 s), 52°C (40 s), 72 ° C (1 min); 3)72°C (10 min). | Palumbi, 1996 |
|  | 16SBr_R | (R) CCGGTCTGAACTCAGATCACGT |  |  |  |
| 28S | 28SC1 | (F) ACCCGCTGAATTTAAGCAT | 809 bp | 1)96°C (4 min); 2) 45 cycles: 94°C (30 s), 48°C (30 s), 72°C (1 min); 3) 72 °C (8 min). | Hassouna et al., 1984 |
|  | 29SD2 | (R) TCCGTGTTTCAAGACGG |  |  |  |

**References for Table S1**

Carr, C.M., Hardy, S.M., Brown, T.M., Macdonald, T.A., Hebert, P.D. (2011). A tri-oceanic perspective: DNA barcoding reveals geographic structure and cryptic diversity in Canadian polychaetes. *PLoS One*, 6, e22232. [https://doi.org/10.1371/ journal. pone.0022232](https://doi.org/10.1371/%20journal.%20pone.0022232)

Hassouna, N., Mithot, B., Bachellerie, J.P. (1984). The complete nucleotide sequence of mouse 28S rRNA gene. Implications for the process of size increase of the large subunit rRNA in higher eukaryotes. *Nucleic Acids Research*, 12, 3563-3583

Palumbi, S.R. (1996). Nucleic acids II: the polymerase chain reaction. *Molecular systematics*, 205-247.
