# Supplementary material for: Molecular diversity within the genus Laeonereis (Annelida, Nereididae) along the west Atlantic coast: paving the way for integrative taxonomy
Source: PeerJ. 2021 May 27;9:e11364. doi: 10.7717/peerj.11364 (PMC8164838; doi:10.7717/peerj.11364)
Supplement: Supplemental Information 2 — Voucher numbers: ZUEC-Poly are from Zoological Museum of University of Campinas (UNICAMP) and MNRJP from Nacional Museum of Rio de Janeiro; * = specimens were used entirely for DNA extraction. [file peerj-09-11364-s002.docx]

**Table S2**

| Taxon | N | State/Country | BOLD code | Voucher | Genbank COI | GenBank 16S | Genbank 28S | Reference |
| --- | --- | --- | --- | --- | --- | --- | --- | --- |
| *Laeonereis culveri* | 3 | Maryland, USA |  |  | MH235843  KU905768  KU905864 |  |  | Aguilar et. al., 2015. |
| *Laeonereis culveri* | 2 | Connecticut, USA | LCCS083-20  LCCS085-20 | * | MT887785 | MT887891  MT887919 |  | Present Study |
| *Laeonereis* sp | 3 | Amapá, BR | LCCS073-20  LCCS074-20  LCCS075-20 | * | MT887732  MT887742  MT887736 | MT887881 |  | Present Study |
|  | 8 | Piauí, BR | LCCS037-19  LCCS038-19  LCCS039-19  LCCS040-19  LCCS041-19  LCCS042-19  LCCS043-19  LCCS044-19 | ZUEC-Poly 21448 | MT887733  MT887741  MT887743  MT887764  MT887752  MT887742  MT887754  MT887775 |  | MT887809  MT887815  MT887827  MT887820  MT887836  MT887833 | Present Study |
|  | 1 | Pará, BR | LCCS079-20 | * | MT887799 |  |  | Present Study |
|  | 7 | Ceará, BR | LCCS045-19  LCCS046-19  LCCS047-19  LCCS048-19  LCCS049-19  LCCS050-19  LCCS051-19 | ZUEC-Poly 21449 | MT887766  MT887734  MT887770  MT887740  MT887776  MT997750  MT887787 |  | MT887829  MT887810  MT887814  MT887834  MT887819  MT887838 | Present Study |
|  | 3 | Bahia, BR | LCCS076-20  LCCS077-20  LCCS078-20 | MNRJP001721 | MT887786  MT887745  MT887773 |  |  | Present Study |
|  | 6 | Rio de Janeiro, BR | LCCS080-20  LCCS081-20  LCCS084-20  LCCS086-20  LCCS087-20  LCCS088-20 | MNRJP001844-001850 | MT887780  MT887793  MT887801 | MT887911  MT887915  MT887918 |  | Present Study |
|  | 27 | Rio de Janeiro, BR |  | MNRJP001844-001850 | \| MH264895 \| \| --- \| \| MH264913 \| \| MH264918 \| \| MH264931 \| \| MH264933 \| \| MH264936 \| \| MH264944 \| \| MH264949 \| \| MH264953 \| \| MH264955 \| \| MH264977 \| \| MH264979 \| \| MH264985 \| \| MH264996 \| \| MH265006 \| \| MH265007 \| \| MH265009 \| \| MH265013 \| \| MH265014 \| \| MH265015 \| \| MH265033 \| \| MH265034 \| \| MH265036 \| \| MH265037 \| \| MH265043 \| \| MH265046 \| \| MH265049 \| |  |  | Seixas et al., 2018. |
|  | 6 | São Paulo, BR | LCCS001-19  LCCS002-19  LCCS003-19  LCCS005-19  LCCS006-19  LCCS095-20 | ZUEC-Poly 21444 | MT606429  MT606437  MT606426  MT606435  MT606430  MT606434 | MT887887  MT887923  MT887882  MT887904  MT887888 | MT887816  MT887841  MT887811  MT887828 | Sampieri et al. 2020 |
|  | 23 | São Paulo, BR | LCCS052-19  LCCS004-19  LCCS007-19  LCCS008-19  LCCS009-19  LCCS010-19  LCCS011-19  LCCS012-19  LCCS013-19  LCCS014-19  LCCS015-19  LCCS016-19  LCCS017-19  LCCS018-19  LCCS019-19  LCCS020-19  LCCS021-19  LCCS022-19  LCCS023-19  LCCS024-19  LCCS025-19  LCCS026-19  LCCS027-19 | ZUEC-Poly 21444  ZUEC-Poly 21445  ZUEC-Poly 21446  ZUEC-Poly 21450 | MT887790  MT887779  MT887728  MT887758  MT887777  MT887737  MT887769  MT887755  MT887751  MT887735  MT887803  MT887730  MT887805  MT887784  MT887798  MT887762  MT887794  MT887731  MT887771  MT887788  MT887796  MT887768  MT887782 | MT887913  MT887877  MT887898  MT887812  MT887884  MT887908  MT887895  MT887893  MT887883  MT887931  MT887879  MT887933  MT887917  MT887928  MT887902  MT887925  MT887880  MT887909  MT887920  MT887927  MT887907  MT887914 | MT887835  MT887823  MT887812  MT887848  MT887808  MT887850  MT887846  MT887826  MT887842  MT887831  MT887839  MT887844  MT887830  MT887837 | Present Study |
|  | 10 | Paraná, BR | LCCS028-19  LCCS029-19  LCCS030-19  LCCS031-19  LCCS032-19  LCCS033-19  LCCS034-19  LCCS035-19  LCCS036-19 | ZUEC-Poly 21447 | MT887783  MT887753  MT887749  MT887767  MT887759  MT887756  MT887789  MT887802  MT887746 | MT887916  MT887894  MT887906  MT887899  MT887896  MT887921  MT887930  MT887889 | MT887821 | Present Study |
|  | 1 | Santa Catarina, BR | LCCS082-20 |  | MT887739 |  |  | Present Study |
|  | 4 | Rio Grande do Sul, BR | LCCS053-19  LCCS054-19  LCCS055-19  LCCS056-19 | ZUEC-Poly 21451 | MT887797  MT887747  MT887792  MT887763 | MT887890  MT887924  MT887903 | MT887845  MT887817 | Present Study |
|  | 6 | Montevideo, UR | LCCS057-19  LCCS058-19  LCCS059-19  LCCS060-19  LCCS061-19  LCCS062-19 | ZUEC-Poly 21452 | MT887778  MT887729  MT887791  MT887738  MT887806  MT887772 | MT887878  MT887922  MT887885  MT887934  MT887910 | MT887878  MT887840  MT887813  MT887851 | Present Study |
|  | 8 | Mar del Plata, AR | LCCS063-19  LCCS064-19  LCCS065-19  LCCS066-19  LCCS068-19  LCCS069-19  LCCS070-19  LCCS071-19 | ZUEC-Poly 21453 | MT887757  MT887748  MT887761  MT887765  MT887795  MT887760  MT887804  MT887774 | MT887897  MT887892  MT887901  MT887905  MT887926  MT887900  MT887932 | MT887822  MT887818  MT887825  MT887843  MT887824  MT887849  MT887832 | Present Study |
| *Ceratocephale cf. loveni* | 1 | Nova Scotia, CAN |  |  |  | MH379973.1 |  | Neal et al., 2018. |
| *Micronereis nanaimoensis* | 1 | British Columbia, CAN |  |  | MF121172.1 |  |  | Dewaard, JR. 2017. |
| *Allita succinea* | 2 | Maryland, USA |  |  | KU906063.1  KU906071.1 | KT959483.1  KT959523.1 | AY210464.1 | Aguilar et al., 2006. |
| *Allita succinea* | 1 | Mar del Plata, AR | LCCS072-19 | ZUEC-Poly 21454 | MT887744 | MT887886 |  | Present Study |

**References for table S2**

**Aguilar R, Ogburn MB, Weight LA, Driskell AC, Hines AH.** Chesapeake Bay Invertebrates, Unpublished, 2015.

**Dewaard, J.R.** Center for Biodiversity Genomics, unpublished, 2017.

**Neal L, Taboada S, Woodall LC.** Slope-shelf faunal link and unreported diversity of Nova Scotia: evidence from polychaete data. *Deep Sea Research – Part I Oceanography*, 138, 72-84, 2018.

**Passamaneck Y, Halanych KM.** Lophotrochozoan phylogeny assessed with LSU and SSU data: Evidence of lophophorate polyphyly. Molecular Phylogenetics and Evolution 40: 20-28, 2006.

**Sampieri, BR et al.** How oogenesis analysis combined with DNA barcode can help to elucidate taxonomic ambiguities: a polychaete study-based approach. Biota Neotropica, 20 (3), 2020. <https://doi.org/10.1590/1676-0611-BN-2020-0959>

**Seixas VC, Paiva PC, Russo CAM. 2018.** Comparative population genetics and demographic history of two polychaeta species suggest that coastal lagoon populations evolve under alternate regimes of gene flow. *Marine Biology* 165: 179.
