# Supplementary material for: Molecular diversity within the genus Laeonereis (Annelida, Nereididae) along the west Atlantic coast: paving the way for integrative taxonomy
Source: PeerJ. 2021 May 27;9:e11364. doi: 10.7717/peerj.11364 (PMC8164838; doi:10.7717/peerj.11364)
Supplement: Supplemental Information 3 — Number of sequences (n); number of variables sites (S); number of haplotypes (h); haplotype diversity (Hd); nucleotide diversity (π). [file peerj-09-11364-s003.docx]

**Table S3**

| Group | n | S | h | Hd | π |
| --- | --- | --- | --- | --- | --- |
| MOTU 1 | 4 | - | 1 | - | - |
| MOTU 2 | 1 | - | 1 | - | - |
| MOTU 3 | 10 | 8 | 5 | 0.822 | 0.00396 |
| MOTU 4 | 8 | 21 | 5 | 0.857 | 0.01625 |
| MOTU 5 | 3 | 3 | 2 | 0.667 | 0.00340 |
| MOTU 6 | 32 | 105 | 11 | 0.808 | 0.02183 |
| MOTU 7 | 55 | 16 | 18 | 0.869 | 0.00284 |
| Total | 113 | 195 | 43 | 0.951 | 0.10282 |
