# Supplementary figures and images for: Molecular diversity within the genus Laeonereis (Annelida, Nereididae) along the west Atlantic coast: paving the way for integrative taxonomy
Source: PeerJ. 2021 May 27;9:e11364. doi: 10.7717/peerj.11364 (PMC8164838; doi:10.7717/peerj.11364)

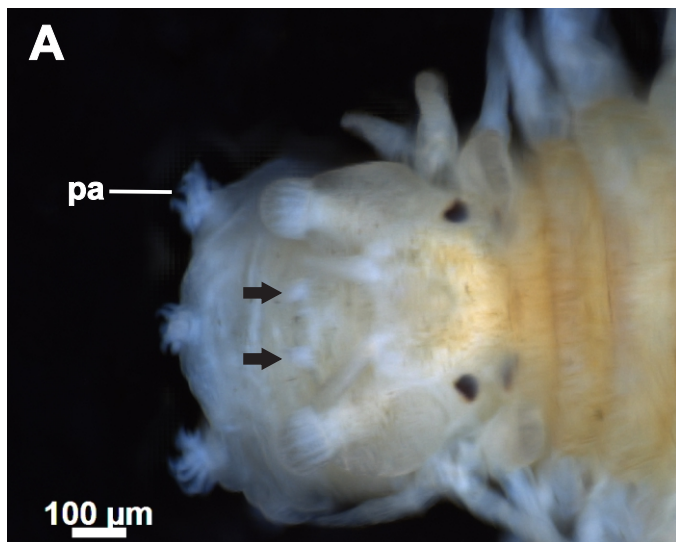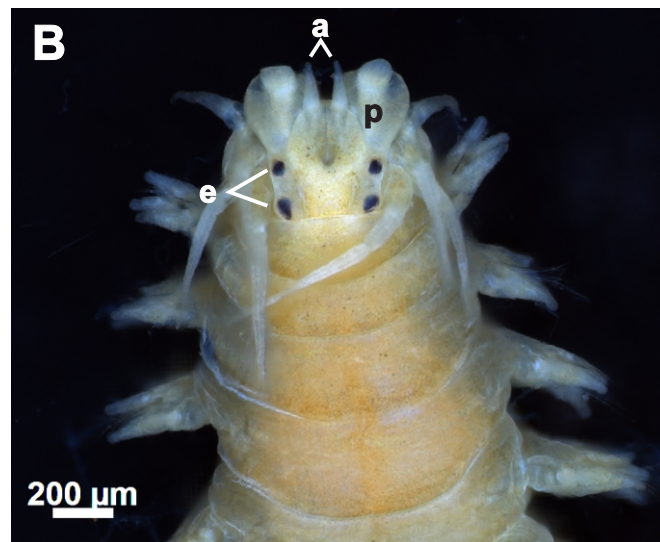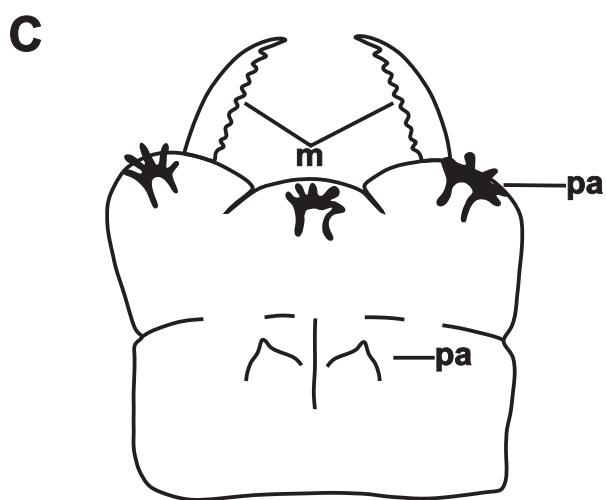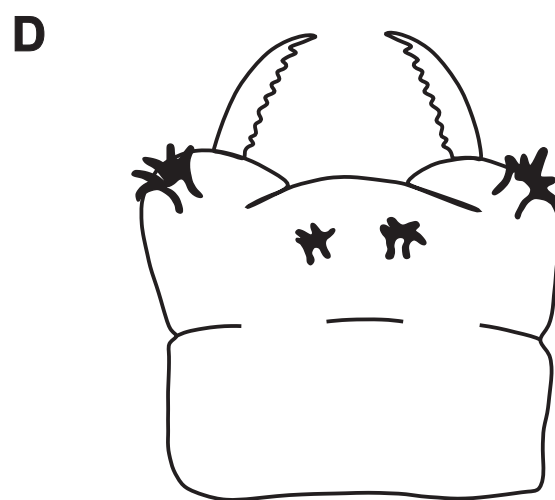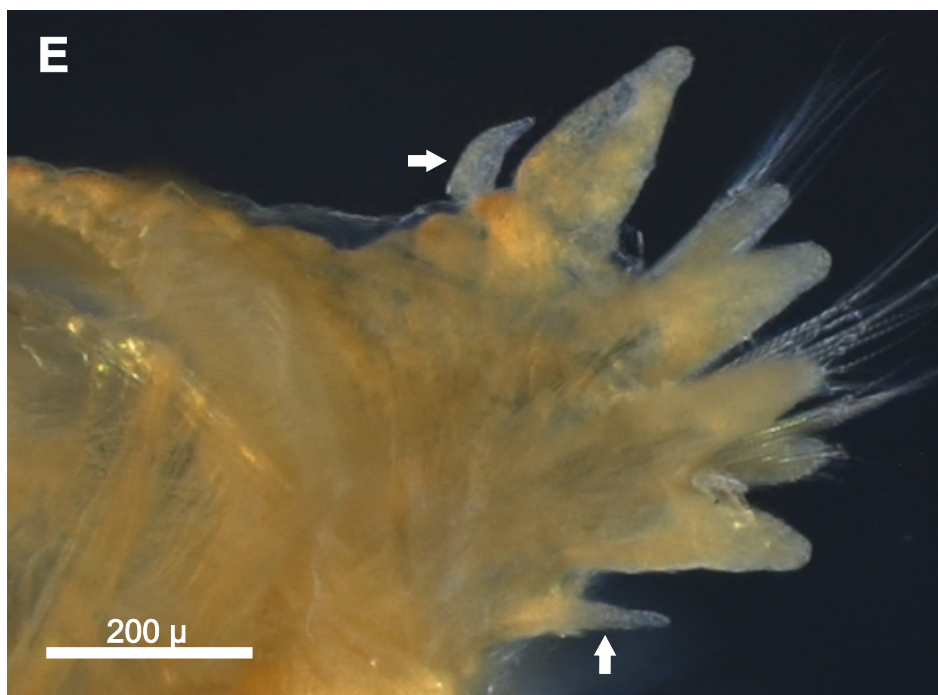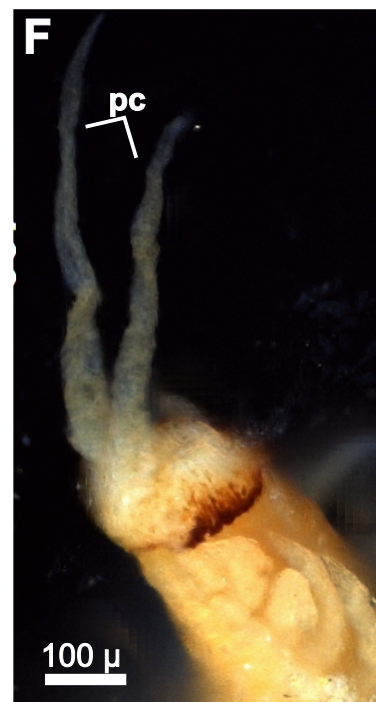

Supplement: Supplemental Information 4 — A-B: Anterior region with details of antenae (a), palps (p) two pair of eyes (e) and everted pharinx with soft papilae (pa; arrows). C-D: Everted pharinx ilustration, with details of the papilae (pa) and mandibles (m). E: Fifth setigers parapodia. F-G: Pigidium. dc: dorsal cirri; L: lingule; pc: pigidial cirri; vc: ventral cirri; white arrows: pigidium rim. [file peerj-09-11364-s004.pdf]
